# The role of rare earth elements and dietary intake in tongue cancer: a mediation analysis in southeast China

**DOI:** 10.3389/fpubh.2023.1058013

**Published:** 2023-04-26

**Authors:** Na Wang, Fengqiong Liu, Yujia Chen, Manling Xie, Bingju Gao, Yu Qiu, Lisong Lin, Bin Shi, Fa Chen, Baochang He

**Affiliations:** ^1^Fujian Provincial Key Laboratory of Environment Factors and Cancer, Department of Epidemiology and Health Statistics, School of Public Health, Fujian Medical University, Fuzhou, Fujian, China; ^2^Department of Oral and Maxillofacial Surgery, The First Affiliated Hospital of Fujian Medical University, Fuzhou, China; ^3^Key Laboratory of Ministry of Education for Gastrointestinal Cancer, Fujian Medical University, Fuzhou, Fujian, China; ^4^Laboratory Center, School of Public Health, The Major Subject of Environment and Health of Fujian Key Universities, Fujian Medical University, Fuzhou, Fujian, China

**Keywords:** rare earth elements, tongue cancer, case-control study, dietary intake, food categories

## Abstract

**Objective:**

The current research aimed to examine how dietary intake and rare earth elements may affect the development of tongue cancer.

**Methods:**

The serum levels of 10 rare earth elements (REEs) in 171 cases and 171 healthy matched controls were measured by inductively coupled plasma mass spectrometry (ICP-MS). The conditional logistic regression was used to examine the relationship between dietary intake, serum levels of 10 REEs, and tongue cancer. Mediation effect and multiplicative interaction analysis were then performed to estimate the potential contribution of REEs in dietary intake associated with tongue cancer.

**Results:**

Compared with the control group, patients with tongue cancer consumed significantly less fish, seafood, fruit, green leafy vegetables, and non-green leafy vegetables, with higher serum praseodymium (Pr), dysprosium (Dy), and lanthanum (La) levels, and lower serum cerium (Ce) and scandium (Sc) levels. The interaction effect was observed between some REEs and food categories. Green vegetables' impact on the risk of tongue cancer is partially attributed to the La and Thorium (Th) elements (*P* < 0.05, the mediated proportion were 14.933% and 25.280%, respectively). The effect of non-green leafy vegetables for tongue cancer mediated via Pr, Dy, and Th (P < 0.05, the mediated proportion were 0.408%, 12.010%, and 8.969%, respectively), and the Sc components in seafood (*P* < 0.05, the mediated proportion was 26.120%) is partly responsible for their influence on the risk of tongue cancer.

**Conclusion:**

The correlation between REEs and dietary intakes for tongue cancer is compact but intricate. Some REEs interact with food intake to influence tongue cancer, while others act as a mediator.

## 1. Introduction

Tongue cancer, including oral tongue cancer (the anterior two-thirds of the tongue in the oral cavity) and the base of tongue cancer (the posterior third of the tongue in the oropharynx), accounts for the most familiar intraoral site for cancer globally (1, 2). Surgery is the primary treatment modality for patients with head and neck cancer, especially tongue cancer. However, larger tongue resection may limit tongue function and quality of later life for patients. The high aspiration rate and complications after surgery may also perplex tongue cancer patients more (3). So, the critical task is to find factors influencing the incidence of tongue cancer. Recent studies showed that tobacco smoking, drinking, poor oral hygiene, and oral disorders might affect the occurrence of tongue cancer in varying degrees (4, 5). Even though dietary intake was closely associated with the tongue, the relationship between dietary intake and tongue cancer was scarcely researched.

According to the International Union of Pure and Applied Chemistry (IUPAC), rare earth elements (REEs) are defined as a group of 17 chemical elements in the periodic table, specifically the 15 lanthanides, scandium, and yttrium (6). REEs are exploited with increasing annual amounts applied to agricultural, medical, zootechnical, and industrial fields (7, 8). REEs are commonly used in agriculture as forage additives and fertilizers and are consumed via the food chain. Approximately 90% of the global REEs are distributed in China, indicating more absorption hazards for the Chinese population (9). Although few studies focus on REEs, several studies still prompt that they might influence cancer. The levels of 15 REEs in lung tumor tissue were demonstrated to be different from those in healthy lung tissue (10). The relationship of REEs with a brain tumor, colorectal, and hepatic cancer was also reported (11). For tongue cancer, a previous study reported that lanthanum and praseodymium ions might affect the activity of the tongue carcinoma Tca8113 cell (12). Interestingly, the nucleotide-binding domain and leucine-rich repeat protein 3 (NLRP3) inflammasome were reported to have been activated by lanthanum, which played a key role in oral squamous cell carcinoma (including tongue cancer) (13, 14). Since the association between other REEs and the risk of tongue cancer is largely unknown, further investigations are needed.

Rare earth elements present in the environment can transfer from soil to edible parts and accumulate continuously (15, 16). Moreover, the accumulated concentration of REEs differed in various food categories, such as cereals, fresh aquatic products, fresh vegetables, fresh meats, and eggs (4). Strong evidence has been provided that a close relationship exists between dietary intake and malignant tumors (especially oral cancer) (17, 18). Thus, verifying whether the relationships between dietary intake and tongue cancer are mediated by the intake of REEs is necessary. This study aimed to evaluate the levels of 10 REEs, namely cerium (Ce), praseodymium (Pr), neodymium (Nd), lanthanum (La), samarium (Sm), europium (Eu), dysprosium (Dy), yttrium (Y), scandium (Sc), and thulium (Th), in serum by inductively coupled plasma mass spectrometry (ICP-MS) and explore the role of REEs and dietary-related factors in tongue cancer and further to access potential intricate effect (interaction and mediation) of them in tongue cancer.

## 2. Material and methods

### 2.1. Sample collection

A case–control study was conducted in the First Affiliated Hospital of Fujian Medical University (Fujian, China), which enrolled patients with primary tongue cancer diagnosed from December 2010 to September 2019. As described previously (19–21), 191 eligible patients were involved if they fulfilled the following criteria: patients who (a) histologically confirmed with primary tongue cancer; (b) aged between 20 and 80 years; and (c) had lived at least 10 years in Fujian Province. The patients were excluded if they fulfilled the following criteria: patients (a) with a long-term intake of dietary supplements; (b) who have experienced neoadjuvant chemotherapy or radiotherapy before surgery; and (c) who are suffering from severe systemic diseases (including liver and renal damage). Control participants without a history of cancer were enlisted throughout the same time frame from the same hospital's health assessment center with the following exclusion criteria: patients (a) who had worked with inorganic materials regularly, such as welders and potters; (b) who aged under 20 or over 80 years old; (c) who had lived in the Fujian Province less than 10 years; and (d) with a long-term intake of dietary supplements. Finally, 1,417 healthy subjects (682 men and 735 women) were recruited. Following propensity score matching (PSM), 171 patients and 171 healthy-matched controls were enrolled in this study.

We have obtained written informed consent from all participants. This research was performed in accordance with the ethical standards of the Helsinki Declaration, and ethics approval was obtained from the Ethics Committees of Fujian Medical University, Fuzhou, China (2011053).

### 2.2. Data collection

General features (age, weight, height, gender, family history of cancer, residence, tobacco smoking, alcohol, and tea drinking) and food categories (fruits, seafood, fish, green leafy vegetables, non-green leafy vegetables, milk, egg, meat, and processed meat consumption) were obtained via face-to-face interview using structured questionnaires. The following options were given to participants when asked about the frequency of each food category: < 1 time per week or not at all, 1–2 times per week, 3–4 times per week, 5–6 times per week, 1 time per day, 2 times per day, and 3 times per day. The questions of food categories were about the diet 1 year before their diagnosis or interview (for controls).

### 2.3. Blood sample collection

Approximately 3–5 ml fasting blood sample of each subject was collected in a trace metal-free tube. The blood samples of cases were collected the day after patients were accepted to the hospital to avoid the impact of any drug treatment or examination. After collection, the blood samples were centrifuged at 1,509 rpm for 10 min at 4°C to separate the serum.

### 2.4. Sample digestion and detection

First, using microwave digestion equipment (PreeKem, China), 200 μl of blood samples were digested with 1 ml of nitric acid (HNO_3_) and 4 ml of ultrapure water. After that, the acid-catching temperature was set to 140°C, and the digestion inner tank was placed on the acid catcher to drive acid until 0.5 ml. After flushing the digestive tube's inner wall more than three times, the flushing solution was added into a volumetric flask, and then ultrapure water was used to create a constant amount of 10 ml. Then, the concentrations of 10 rare earth elements (Ce, Pr, Nd, Sm, Eu, Dy, Sc, La, Y, and Th) were measured by inductively coupled plasma mass spectrometry (ICP-MS, NexION 350X; Perkin-Elmer, USA). The instrument parameters are shown in Supplementary Table 1. The limit of detection and the percentage below LOD (%) are described in Supplementary Table 2, and REEs were enrolled for further analysis with a detection rate above 50%.

### 2.5. The analytic quality controls

We used human hair powder (GBW07601a, China) as a standard reference material for maintaining method performance for quality control. The spike-and-recovery test also showed the validity of measurement (range: 80–105%) (Supplementary Table 2). For every batch, at least two standard reference materials and two blanks were measured. The variation coefficients were < 5%, and 12.5% of each batch's samples were repeated.

### 2.6. Statistical analysis

A 1:1 propensity score matching (PSM) (22) was used to balance the potential confounding (age, gender, family history of cancer, residence, Body Mass Index (BMI), tea and alcohol drinking, and tobacco smoking) between case and control groups with the nearest-neighbor matching approach (maximum caliper distance, 0.02). The group differences before and after PSM were evaluated using the chi-square test or *t*-test. The distribution state of each REE was tested by the Shapiro–Wilk test method, while the Wilcoxon rank sum test was used in the case of non-normal distribution. The associations of each feature and each REE with tongue cancer were tested by univariate and multivariate conditional logistic regression based on the “stats” package (R software). Odds ratios (ORs) and 95% confidence intervals (CIs) were presented. Then, we evaluated the interaction effect of food intake and each REE in tongue cancer, the interaction term was multiplied by food categories, and each REE was included in the multivariable conditional logistic regression model. If the interaction term was significantly associated with tongue cancer, a dummy variable regression analysis (23) would then further be performed. Finally, based on the “mediation” package (R software), the mediation analysis was performed. All analyses were based on R software version 4.1.3. All *p*-values were two-sided, and a *P* < 0.05 was considered statistically significant.

## 3. Results

### 3.1. Patients' characteristics

The comparisons of general characteristics between case and control groups before and after PSM are presented in Supplementary Table 3. Age, gender, residence, family history of cancer, BMI, smoking, alcohol, and tea-drinking status distributions were different (*P* < 0.05), but the distributions of most general characteristics were uniform and comparable between the case and control groups after PSM (*P* > 0.05).

### 3.2. Relationship between dietary intake and tongue cancer

In total, 10 food categories from a food frequency questionnaire were used to assess the dietary intake of enrolled subjects. An increased diet of fish, seafood, fruit, green leafy vegetable, and non-green leafy vegetable was closely associated with decreased risk of tongue cancer and the adjusted OR (95% CI) which were 0.343 (0.181, 0.651), 0.270 (0.146, 0.497), 0.326 (0.175, 0.607), 0.304 (0.160, 0.580), and 0.141 (0.067, 0.295), respectively (Table 1, Model-1). The independent link between dietary intake and tongue cancer after further adjusting for REEs was also investigated in Table 1 (Model-2).

**Table 1:** Food categories of enrolled subjects.

						**Model-1**		**Model-2**	
**Variables**	**Categories**	**Control**	**Case**	χ^2^	**P** ^*^	***OR 95%CI***	***P***	***OR 95%CI***	***P***
Meat				1.42	0.233				
	< 1 time/day	86	98					
	≥1 time/day	85	73			0.878 (0.484, 1.593)	0.670	0.772 (0.462, 1.672)	0.610
Processed meat				1.79	0.181			
	< 1 time/week	152	160						
	≥1 time/week	19	11			0.439 (0.134 ,1.433)	0.170	0.375 (0.155, 0.546)	0.270
Fish				9.68	**0.002**			
	< 2 times/week	101	129						
	≥2 times/week	70	42			0.343 (0.181 ,0.651)	**0.001**	0.221 (0.128, 0.276)	**0.006**
Seafood				27.08	**< 0.001**			
	< 1 time/week	55	104						
	≥1 time/week	116	67			0.270 (0.146, 0.497)	**< 0.001**	0.361 (0.217, 0.518)	**0.045**
Milk				5.95	**0.015**			
	< 1 time/week	93	116					
	≥1 time/week	78	55			0.623 (0.346, 1.119)	0.110	0.664 (0.418, 1.289)	0.380
Egg				0.61	0.434			
	< 2 times/week	103	111						
	≥2 times/week	68	60			0.862 (0.475, 1.563)	0.620	0.602 (0.377, 1.099)	0.280
Green leafy vegetables				14.95	**< 0.001**			
	< 2 times/day	50	86					
	≥2 times/day	121	85			0.304 (0.160, 0.580)	**< 0.001**	0.203 (0.120, 0.249)	**0.003**
Non-green leafy vegetables				43.53	**< 0.001**			
	< 2 times/day	56	118						
	≥2 times/day	115	53			0.141 (0.067, 0.295)	**< 0.001**	0.055 (0.026, 0.058)	**< 0.001**
Fruit				26.87	**< 0.001**			
	< 2 times/week	86	133					
	≥2 times/week	85	38			0.326 (0.175, 0.607)	**< 0.001**	0.104 (0.054, 0.116)	**0.001**
Pickled food				0.01	0.904				
	< 1 time/week	124	122					
	≥1 time/week	47	49			0.938 (0.756, 1.164)	0.560	0.951 (0.785, 2.461)	0.790
Total		171	171						

^*^P values of Chi-square test. Model-1: conditional logistic regression adjusted Gender, age, residence, family history of cancers, BMI, tobacco smoking, tea, and alcohol drinking. Model-2: conditional logistic regression adjusted Gender, age, residence, family history of cancers, BMI, tobacco smoking, tea, and alcohol drinking and each REE. Bolded values indicate statistical significance at *p* < 0.05.

### 3.3. Relationship between rare earth elements and tongue cancer

**Figure 1:**
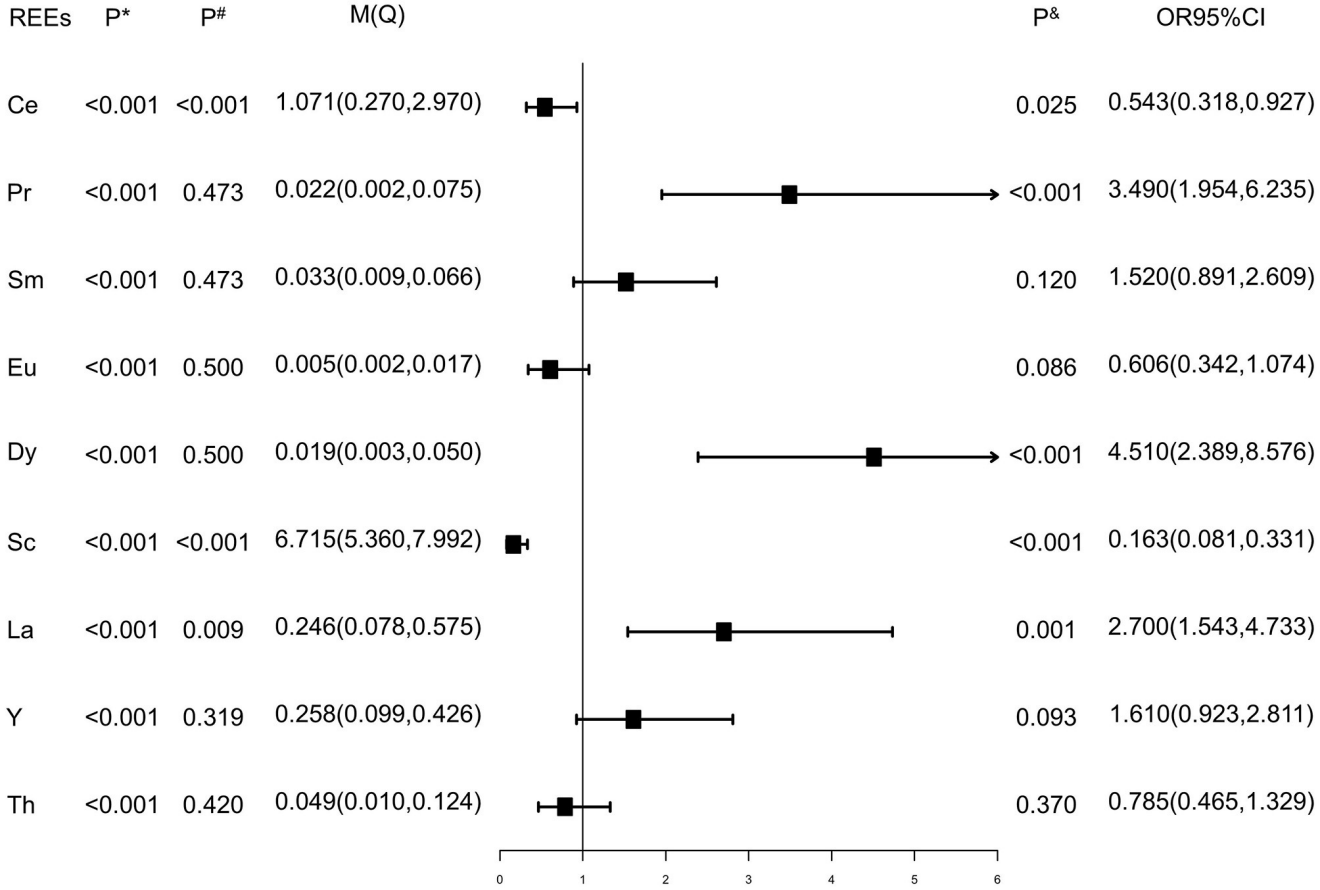
Rare earth elements of enrolled subjects. P*: P values of Shapiro-Wilk test. P^#^: P values of Wilcoxon rank sum test. P^&^: P values of logistic regression adjusted age, gender, residence, family history of cancers, BMI, tobacco smoking, tea, and alcohol drinking. M(Q): Median (quartile25, quartile75), μg/L.

**Figure 2:**
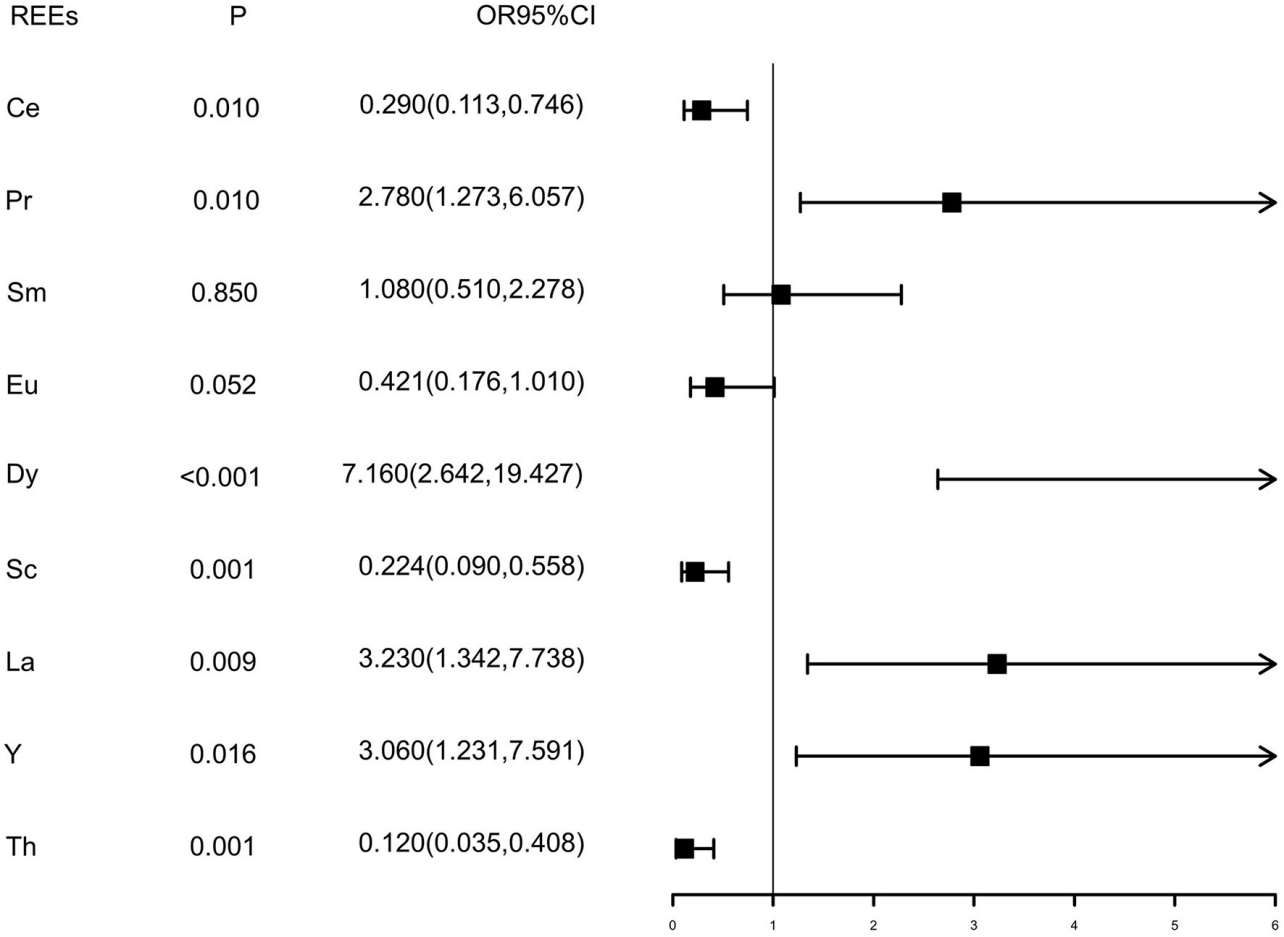
Multivariate conditional logistic regression results of REE elements. Pd: P-values of conditional logistic regression adjusted age, gender, residence, family history of cancer, BMI and alcohol drinking, tobacco smoking, tea drinking, and fruits, seafood, fish, green leafy vegetables, non-green leafy vegetables, milk, egg, meat, processed meat, and pickled food consumption.

Values of each REE below the detection limit were replaced by half of the detection limits (15). As shown in Figure 1, the Wilcoxon rank sum test showed that the distributions of Ce, Sc, and La were different between case and control groups (*P*^#^ < 0.05), while the distributions of Pr, Sm, Eu, Y, and Th were similar between the two groups (*P*^#^ > 0.05). Elements were dichotomized into low and high groups based on the median concentration value of healthy controls, and the cutoff values are presented in Supplementary Table 4. Inverse relationships were found between serum Ce, Sc, and tongue cancer [OR and 95% CI were 0.543 (0.318, 0.927) and 0.163 (0.081, 0.331), respectively], while direct relationships were found between Pr, Dy, La, and tongue cancer [OR and 95% CI were 3.490 (1.954, 6.235),4.510 (2.389, 8.576), and 2.700 (1.543, 4.733), respectively] after adjusting for gender, residence, family history of cancer, BMI, tea drinking, tobacco smoking, and alcohol drinking (Figure 1). As results presented in Figure 2, we found that after additional adjusting for dietary intakes, the relationships between some REEs and tongue cancer were changed.

### 3.4. Interaction effect of the REEs and dietary intake for tongue cancer

Significant interaction effects were observed between serum levels of La and dietary intake of non-green leafy vegetables; between serum levels of Ce and Pr and dietary intake of green leafy vegetables; and between serum levels of Eu and dietary intake of seafood or non-green leafy vegetables for tongue cancer (Table 2 all P_interaction_ < 0.05). The results of the stratified analysis for the REEs and food categories were further discussed and are presented in Table 2.

**Table 2:** The combined effect of the REE and dietary intakes for tongue cancer.

**Variables**	**Food categories**	**β**	**OR95%CI**	***P***	***P_*interaction*_***
**Eu**	**Seafood**				**0.036**
Low	< 1 time/week				
High	< 1 time/week	−1.831	0.160 (0.042, 0.606)	**0.007**	
Low	≥1 time/week	−2.345	0.096 (0.024, 0.389)	**0.001**	
High	≥1 time/week	−2.351	0.095 (0.023, 0.388)	**0.001**	
**Eu**	**Non-green leafy vegetables**				**0.017**
Low	< 2 times/day				
High	< 2 times/day	0.159	1.170 (0.342, 4.022)	0.800	
Low	≥2 times/day	−1.090	0.336 (0.080, 1.409)	0.140	
High	≥2 times/day	−3.207	0.041 (0.008, 0.214)	**< 0.001**	
**Ce**	**Green leafy vegetables**				**0.039**
Low	< 2 times/day				
High	< 2 times/day	−0.084	0.920 (0.220, 3.839)	0.910	
Low	≥2 times/day	1.910	6.750 (1.110, 41.053)	**0.038**	
High	≥2 times/day	−0.224	0.799 (0.189, 3.375)	0.760	
**Pr**	**Green leafy vegetables**				**0.030**
Low	≥2 times/day				
High	≥2 times/day	0.575	6.160 (3.474, 10.952)	**0.002**	
Low	< 2 times/day	0.736	1.770 (0.848, 3.697)	0.440	
High	< 2 times/day	0.739	1.670 (0.798, 3.500)	0.490	
**La**	**Non-green leafy vegetables**				**0.046**
Low	< 2 times/day				
High	< 2 times/day	1.979	7.230 (2.096, 24.969)	**0.002**	
Low	≥2 times/day	−1.658	0.191 (0.046, 0.788)	**0.022**	
High	≥2 times/day	−1.268	0.281 (0.063, 1.255)	0.097	

P: The P values of stratified analysis adjusted age, gender, residence, family history of cancers, BMI and alcohol drinking, tobacco smoking, drinking tea, and fruits, seafood, fish, green leafy vegetables, non-green leafy vegetables, milk, egg, meat, processed meat, pickled food consumption. *Pinteraction*: The P values of interaction analysis of the REE and food categories for tongue cancer adjusted age, gender, residence, family history of cancers, BMI and alcohol drinking, tobacco smoking, tea drinking, and fruits, seafood, fish, green leafy vegetables, non-green leafy vegetables, milk, egg, meat, processed meat, pickled food consumption. Bolded values indicate statistical significance at *p* < 0.05.

### 3.5. Mediated effect of the REEs and dietary intakes for tongue cancer

We selected food categories that were significantly related to tongue cancer and assessed the potential mediation effect of each REE in the relationships between the food categories and the risk of tongue cancer. The total effect, direct effect, mediated effect, and mediated proportions of each REE are shown in Table 3. We observed that some serum REEs were related to food intake (P^*^ < 0.05). Pr, Dy, and Th act partially as mediators between intake of non-green leafy vegetables and risk of tongue cancer (*P* < 0.05, the mediated proportion were 0.408%, 12.010%, and 8.969%, respectively). La and Th perform as mediators between intake of green leafy vegetables and tongue cancer with the mediated proportions of 14.933% and 25.280%, respectively. The relation between seafood consumption and tongue cancer was mediated by Sc with a mediated proportion of 26.120%.

**Table 3:** The mediation effect of the REE and food categories for tongue cancer.

**Variables**	**Total effect**	**Direct effect**	**Mediated effect**	**Proportion mediated (%)**	***P***	P^*^
**Ce**						
Seafood	−0.148 (−0.211, −0.070)	−0.150 (−0.213, −0.080)	0.003 (−0.006, 0.020)	17.267	0.700	0.375
Fish	−0.032 (−0.126, 0.070)	−0.036 (−0.138, 0.080)	0.004 (−0.006, 0.020)	3.048	0.540	0.248
Fruit	−0.164 (−0.242, −0.080)	−0.167 (−0.244, −0.080)	0.003 (−0.007, 0.010)	0.549	0.740	0.783
Green leafy vegetables	0.071 (−0.074, 0.180)	0.078 (−0.071, 0.190)	−0.007 (−0.026, 0.000)	4.750	0.260	**0.044**
Non-green leafy vegetables	−0.231 (−0.267, −0.180)	−0.233 (−0.273, −0.190)	0.002 (−0.005, 0.010)	0.408	0.700	0.291
**Pr**						
Seafood	−0.145 (−0.222, −0.060)	−0.121 (−0.207, −0.040)	−0.024 (−0.060, 0.000)	15.820	0.100	**0.015**
Fish	−0.038 (−0.139, 0.080)	−0.038 (−0.138, 0.090)	0.000 (−0.030, 0.030)	1.417	0.980	0.937
Fruit	−0.167 (−0.251, −0.060)	−0.163 (−0.248, −0.070)	−0.004 (−0.029, 0.020)	2.809	0.760	0.635
Green leafy vegetables	0.062 (−0.080, 0.160)	0.041 (−0.105, 0.150)	0.021 (−0.005, 0.040)	23.944	0.160	0.109
Non-green leafy vegetables	−0.232 (−0.265, −0.190)	−0.202 (−0.236, −0.150)	−0.030 (−0.057, −0.010)	0.408	**< 0.001**	**0.003**
**Sm**						
Seafood	−0.138 (−0.201, −0.040)	−0.137 (−0.200, −0.040)	−0.002 (−0.015, 0.010)	0.571	0.800	0.637
Fish	−0.030 (−0.145, 0.070)	−0.032 (−0.143, 0.070)	0.002 (−0.007, 0.020)	0.092	0.660	0.683
Fruit	−0.163 (−0.230, −0.070)	−0.165 (−0.230, −0.070)	0.001 (−0.005, 0.010)	0.410	0.820	0.592
Green leafy vegetables	0.048 (−0.090, 0.160)	0.041 (−0.095, 0.160)	0.007 (−0.005, 0.030)	2.522	0.240	0.098
Non-green leafy vegetables	−0.231 (−0.269, −0.200)	−0.225 (−0.262, −0.190)	−0.007 (−0.024, 0.000)	7.417	0.420	**0.007**
**Eu**						
Seafood	−0.152 (−0.208, −0.070)	−0.156 (−0.211, −0.070)	0.004 (−0.007, 0.020)	2.327	0.440	0.358
Fish	−0.021 (−0.128, 0.100)	−0.028 (−0.137, 0.100)	0.007 (−0.006, 0.030)	3.769	0.340	0.131
Fruit	−0.162 (−0.227, −0.060)	−0.160 (−0.226, −0.060)	−0.002 (−0.016, 0.010)	0.550	0.740	0.359
Green leafy vegetables	0.069 (−0.049, 0.170)	0.078 (−0.044, 0.180)	−0.010 (−0.030, 0.000)	9.385	0.120	0.090
Non-green leafy vegetables	−0.228 (−0.264, −0.190)	−0.235 (−0.271, −0.200)	0.007 (−0.006, 0.020)	2.878	0.240	0.116
**Dy**						
Seafood	−0.147 (−0.210, −0.040)	−0.135 (−0.198, −0.030)	−0.012 (−0.036, 0.010)	7.200	0.260	0.123
Fish	−0.042 (−0.134, 0.060)	−0.046 (−0.140, 0.050)	0.005 (−0.027, 0.040)	0.365	0.720	0.501
Fruit	−0.157 (−0.243, −0.070)	−0.153 (−0.232, −0.070)	−0.004 (−0.027, 0.020)	2.049	0.760	0.613
Green leafy vegetables	0.054 (−0.070, 0.160)	0.029 (−0.091, 0.140)	0.025 (−0.001, 0.050)	27.974	0.080	**0.025**
Non-green leafy vegetables	−0.226 (−0.264, −0.180)	−0.198 (−0.243, −0.140)	−0.028 (−0.047, −0.010)	12.010	**< 0.001**	**< 0.001**
**Sc**						
Seafood	−0.146 (−0.225, −0.060)	−0.106 (−0.175, −0.030)	−0.040 (−0.069, −0.010)	26.120	**< 0.001**	**0.004**
Fish	−0.039 (−0.126, 0.080)	−0.016 (−0.105, 0.090)	−0.023 (−0.060, 0.010)	31.410	0.120	0.176
Fruit	−0.166 (−0.242, −0.070)	−0.141 (−0.221, −0.040)	−0.025 (−0.057, 0.000)	15.590	0.140	0.074
Green leafy vegetables	0.059 (−0.074, 0.160)	0.077 (−0.045, 0.170)	−0.018 (−0.064, 0.020)	11.280	0.360	0.192
Non-green leafy vegetables	−0.231 (−0.270, −0.180)	−0.231 (−0.277, −0.180)	0.000 (−0.024, 0.030)	1.000	0.123	0.995
**La**						
Seafood	−0.140 (−0.207, −0.070)	−0.127 (−0.193, −0.050)	−0.013 (−0.031, 0.000)	8.360	0.100	0.044
Fish	−0.028 (−0.149, 0.070)	−0.031 (−0.161, 0.070)	0.004 (−0.018, 0.030)	1.693	0.680	0.570
Fruit	−0.166 (−0.244, −0.060)	−0.166 (−0.247, −0.070)	0.000 (−0.018, 0.020)	< 0.001	0.140	0.980
Green leafy vegetables	0.057 (−0.080, 0.160)	0.041 (−0.097, 0.150)	0.016 (0.001, 0.040)	14.933	**0.020**	**0.044**
Non-green leafy vegetables	−0.227 (−0.265, −0.190)	−0.214 (−0.251, −0.170)	−0.013 (−0.032, 0.000)	5.247	0.060	**0.006**
**Y**						
Seafood	−0.147 (−0.205, −0.060)	−0.148 (−0.207, −0.070)	0.001 (−0.006, 0.010)	0.388	0.680	0.564
Fish	−0.030 (−0.136, 0.080)	−0.033 (−0.138, 0.080)	0.002 (−0.008, 0.010)	0.780	0.820	0.114
Fruit	−0.159 (−0.236, −0.050)	−0.160 (−0.234, −0.050)	0.001 (−0.007, 0.010)	0.149	0.820	0.839
Green leafy vegetables	0.065 (−0.062, 0.150)	0.063 (−0.060, 0.150)	0.002 (−0.008, 0.010)	1.205	0.740	0.304
Non-green leafy vegetables	−0.227 (−0.262, −0.180)	−0.225 (−0.265, −0.180)	−0.002 (−0.011, 0.010)	0.418	0.620	0.228
**Th**						
Seafood	−0.144 (−0.212, −0.060)	−0.151 (−0.216, −0.070)	0.007 (−0.006, 0.020)	4.332	0.420	0.293
Fish	−0.037 (−0.134, 0.070)	−0.032 (−0.143, 0.070)	0.002 (−0.007, 0.020)	0.360	0.660	0.922
Fruit	−0.174 (−0.242, −0.090)	−0.177 (−0.243, −0.090)	0.004 (−0.016, 0.020)	1.582	0.660	0.594
Green leafy vegetables	0.063 (−0.076, 0.150)	0.087 (−0.057, 0.170)	−0.023 (−0.043, 0.000)	25.280	**0.020**	**0.003**
Non-green leafy vegetables	−0.233 (−0.270, −0.190)	−0.254 (−0.295, −0.200)	0.021 (0.002, 0.040)	8.969	**0.040**	**< 0.001**

P: The P values of mediated effect for tongue cancer adjusted for age, gender, residence, family history of cancers, BMI and alcohol drinking, tobacco smoking, tea drinking, and fruits, seafood, fish, green leafy vegetables, non-green leafy vegetables, milk, egg, meat, processed meat, pickled food consumption. P^*^: The P values of the relations of the REE and food categories adjusted for age, gender, residence, family history of cancers, BMI and alcohol drinking, tobacco smoking, tea drinking, and fruits, seafood, fish, green leafy vegetables, non-green leafy vegetables, milk, egg, meat, processed meat, pickled food consumption. Bolded values indicate statistical significance at *p* < 0.05.

## 4. Discussion

The relationship of diatery intake or REEs with varied types of cancer [oral and pharyngeal cancer (24), breast cancer (25, 26), thyroid cancer (27), pancreatic cancer (28), and lung cancer (10)] has been reported, but hitherto, their role and potential interaction in tongue cancer have still not been elucidated. After minimizing the potential confounding effects of PSM, our study supported that both serum REE levels and food categories were associated with the risk of tongue cancer, and potential multiplicative interaction and mediated effect existed between the two parameters.

Fruit, fish, seafood, green leafy vegetables, and other vegetable intake have been reported to be inversely related to oral squamous cell carcinoma, the pharynx, and larynx cancer risks, which were also observed in our study, and it may be ascribed to the abundant potential anticarcinogenic agents present in foods, such as carotenoids, dietary fiber, n-3 fatty acid, and vitamins C and E (29–32). However, the epidemiological evidence for a protective effect of food categories against cancer was inconsistent. A previous meta-analysis assessed the effect of fish intake on the risk of oral cancer and found a positive relationship in European populations rather than in other populations (33). The significant association between a higher intake of processed meat and the increased risk of oral cancer and oropharynx cancer has been suggested by numerous studies, partly owing to the potent mutagens caused during preservation or meat processing and high-temperature cooking [such as polycyclic aromatic hydrocarbons (PAHs) and N-nitroso compounds (NOCs)] (34–36). These mutagens will bind with DNA and produce PAH-DNA adducts, causing a growing risk tendency for many types of cancer (37, 38). However, other academics believed there was insufficient epidemiological evidence to substantiate an independent positive link between them (39). Existing research elucidates the role of food categories for oral cavity and pharynx cancer clearly, but few reports directly explore the relationship between dietary intake and tongue cancer.

Our research revealed that the serum levels of Pr, Dy, and La increased the risk of tongue cancer, whereas Ce and Sc reduced it. Dysregulation of cellular apoptosis was a key promotion of tumorigenesis. Although no direct evidence implicated that some REEs may exert an effect on cell apoptotic, exposure to REEs could increase telomerase activity, which may be associated with DNA replication and cell apoptosis (40). Experiments proved that CeO_2_ will make more cells stay in the G1 phase and decrease the production of reactive oxygen to inhibit the proliferation of tumor cells (41). Cerium oxide nanoparticles were also reported to inhibit the proliferation and promote the apoptosis of tumor cells selectively (42). Europium oxide nanorods metallocene complexes with scandium characterized anti-proliferative activity in several cancer cell lines, including triple-negative breast cancer cell line (MDA.MB231) and non-hormone sensitive prostate cancer cell line (DU145), which supported that Sc may perform as a protective factor in cancer (43). Clinical studies on Eu and Th have been conducted, which using them as new target therapies for a variety of malignancies (44, 45). To the best of our knowledge, Pr and La would induce adverse developmental effects in zebrafish embryos (especially neural and cardiovascular development) (46). Dy was shown to increase antioxidant defenses, oxidative stress, and cellular damage in mussels with a dose-dependent response, which may support our results to a certain degree (47).

Many reports highlighted that some pollutants including contamination of heavy metals and organic pollutants may pose a health risk (especially cancer) to humans via the food chain (48–51), but the REEs were almost unheeded, although it is an indisputable fact that those are widespread in various foods. Our study found that after adjusting for food categories, the effects of Y and Th for tongue cancer were covered up. Meanwhile, the ORs of food categories were modified after adjusting for REEs. This suggests that dietary intake and REEs interact intricately in tongue cancer. So, the latent combined or mediated effect of dietary intake and serum REEs for tongue cancer was then investigated. In this study, several serum REEs were associated with the intake of some food. The further stratified analysis results showed that people with lower serum levels of Eu and who consume less seafood have a higher risk of tongue cancer than others, indicating that the joint effect between serum Eu and seafood consumption under the multiplicative model was greater than expected. When people have higher serum Eu levels, the protective impact of intake of more non-green leafy vegetables is increased (OR was 0.041). The risk effect of tongue cancer was stronger in the group with low serum Ce level and intake of high green leafy vegetables or the group with high serum Pr level and intake of high green leafy vegetables. Compared with populations who eat fewer non-green leafy vegetables and have lower serum La levels, those with higher serum La levels have an increased risk (OR was 7.230), while those who eat more non-green leafy vegetables have a decreased risk (OR was 0.191). Those indicated that the interactions of the levels of some serum REEs and intake of some food categories played important roles in tongue cancer. The changes in tongue cancer risk due to dietary changes were influenced by the levels of some serum REEs.

In order to know whether dietary control may limit the intake of some REEs and further induce or inhibit tongue cancer, we tested the potential mediate effects of REEs. Our study suggests that green vegetables' impact on the risk of tongue cancer is partially attributed to the La and Th elements. The Pr, Dy, and Th components in non-green leafy vegetables and the Sc components in seafood are partly responsible for their influence on the risk of tongue cancer. According to a Chinese study in 2012, the concentrations of Ce, Dy, Y, Nd, and La in some food categories were higher than the concentrations of other REEs, whereas green vegetables and aquatic products have higher quantities of total rare earth element oxides than other food categories (52). Another report also noted that vegetables in mining regions contain higher levels of some REEs (including La, Pr, and Dy), and the southeast province of China investigated in our study is one of the key mining regions (26, 53). A study found that marine algae are effective natural adsorbents for some REEs, particularly for the Sc element (54). As the Fujian province is a significant coastal region, its residents would consume more marine algae than those in other locations, increasing their exposure to the Sc element. These studies help to explain some of our findings.

Our study creatively explored the mediated effect and multiplicative interaction of food categories and REEs on the development of tongue cancer. The findings of this research will offer direction for daily food and future mechanistic research on the pathophysiology of tongue cancer. However, the limitation also should not be ignored, due to the relatively weak causal reference of the case–control studies with a small sample, we cannot elucidate the causal relationship between REEs and tongue cancer. Thus, more direct epidemiological evidence from a large-scale prospective study needs to be collected in future studies. Then, the cases enrolled in the study were only from one hospital, and the dietary information was recalled by each participant (precise and explicit quantifications are not available); the bias cannot be avoided. Furthermore, intentional drug usage history concealment by participants may have an impact on the levels of serum REEs. In addition to that, though many measures were taken by us to reduce the difference, we cannot deny the possibility that the case and control are from two different populations according to the present eligibility criteria. Finally, the concentrations of elements may change due to exposure to air, water, cooking, and storage techniques; that is the information we cannot access.

## 5. Conclusion

The correlation between REEs and dietary intake for tongue cancer is compact but intricate; the change in dietary intake may change the serum levels of several REEs and further influence the risk of tongue cancer. The joint effect between REEs and food categories in tongue cancer should not be overlooked. Further prospective studies are still needed in validating our findings and exploring the underlying mechanism.

## Data availability statement

The raw data supporting the conclusions of this article will be made available by the authors, without undue reservation.

## Ethics statement

This research was performed in accordance with the ethical standards of the Helsinki Declaration, and the ethical approval was obtained from the Ethics Committees of Fujian Medical University, Fuzhou, China (2011053). The patients/participants provided their written informed consent to participate in this study. Written informed consent was obtained from the individual(s) for the publication of any potentially identifiable images or data included in this article.

## Author contributions

NW: formal analysis, writing—original draft, and writing—review and editing. FL and MX: investigation and writing—review and editing. YC: methodology and writing—review and editing. BG: validation and writing—review and editing. YQ, LL, and BS: resources and writing—review and editing. BH: conceptualization, funding acquisition, and writing—review and editing. FC: conceptualization, funding acquisition, formal analysis, and writing—review and editing. All authors contributed to the article and approved the submitted version.
