# Supplementary material for: The role of rare earth elements and dietary intake in tongue cancer: a mediation analysis in southeast China
Source: Front Public Health. 2023 Apr 26;11:1058013. doi: 10.3389/fpubh.2023.1058013 (PMC10169683; doi:10.3389/fpubh.2023.1058013)
Supplement: Supplementary file 1 [file Table_1.DOCX]

**Contents**

**Supplement Table 1** Setting parameters for the ICP-MS method.

**Supplement Table 2** The limit of detection (LOD) and the percentage below LOD (%) of the REEs

**Supplement Table 3** General characteristics of case and control subjects before and after PSM

**Supplement Table 4** The cut-off values of rare earth elements.

| **Supplement Table 1** Setting parameters for the ICP-MS method. | |
| --- | --- |
| Operating Conditions | Values |
| RF Power (P/W) | 1300 |
| Auxiliary gas flow (q/(L/min)) | 1.10 |
| Nebulizer gas flow (q/(L/min)) | 0.96 |
| Plasma Gas flow (q/(L/min)) | 15 |
| Sweeps per reading | 20 |
| Replicates | 3 |

| **Supplement Table 2** The limit of detection (LOD) and spike recovery of the REEs | | | | | | | | | | | | | | | |
| --- | --- | --- | --- | --- | --- | --- | --- | --- | --- | --- | --- | --- | --- | --- | --- |
| Elements | | | LOD (μg/L) | | Percentage below LOD (%) | | | | | Spiked level (μg/L) | | | Recovery (%) | |  |
| Cerium (Ce) | | | 0.017 | | 4.70 | | | | | 0.02 | | | 105% | |  |
|  | | |  | |  | | | | | 0.1 | | | 92% | |  |
|  | | |  | |  | | | | | 0.5 | | | 88% | |  |
| Praseodymium (Pr) | | | 0.005 | | 29.17 | | | | | 0.02 | | | 90% | |  |
|  | | |  | |  | | | | | 0.1 | | | 94% | |  |
|  | | |  | |  | | | | | 0.5 | | | 91% | |  |
| Neodymium (Nd) | | | 0.027 | | 58.87 | | | | | 0.02 | | | 104% | |  |
|  | | |  | |  | | | | | 0.1 | | | 94% | |  |
|  | | |  | |  | | | | | 0.5 | | | 91% | |  |
| Samarium (Sm) | | | 0.019 | | 34.41 | | | | | 0.02 | | | 105% | |  |
|  | | |  | |  | | | | | 0.1 | | | 94% | |  |
|  | | |  | |  | | | | | 0.5 | | | 90% | |  |
| Europium (Eu) | | | 0.003 | | 40.46 | | | | | 0.02 | | | 95% | |  |
|  | | |  | |  | | | | | 0.1 | | | 95% | |  |
|  | | |  | |  | | | | | 0.5 | | | 91% | |  |
| Dysprosium (Dy) | | | 0.006 | | 39.38 | | | | | 0.02 | | | 95% | |  |
|  | | |  | |  | | | | | 0.1 | | | 92% | |  |
|  | | |  | |  | | | | | 0.5 | | | 89% | |  |
| Scandium (Sc) | | | 0.203 | | 0 | | | | | 0.02 | | | 80% | |  |
|  | | |  | |  | | | | | 0.1 | | | 103% | |  |
|  | | |  | |  | | | | | 0.5 | | | 83% | |  |
| Lanthanum (La) | | | 0.010 | | 6.99 | | | | | 0.02 | | | 90% | |  |
|  | | |  | |  | | | | | 0.1 | | | 94% | |  |
|  | | |  | |  | | | | | 0.5 | | | 90% | |  |
| Yttrium (Y) | | | 0.008 | | 11.69 | | | | | 0.02 | | | 85% | |  |
|  | | |  | |  | | | | | 0.1 | | | 85% | |  |
|  | | |  | |  | | | | | 0.5 | | | 81% | |  |
| Thorium (Th) | | | 0.007 | | 20.56 | | | | | 0.02 | | | 90% | |  |
|  | | |  | |  | | | | | 0.1 | | | 93% | |  |
|  | | |  | |  | | | | | 0.5 | | | 87% | |  |
| **Supplement Table 3** General characteristics of case and control subjects before and after PSM | | | | | | | | | | | | | | | |
| Various | Categories | Before PSM | | | | | |  | After PSM | | | | | | |
|  |  | Control | | Case | | χ²/t | *P* |  | Control | | Case | χ²/t | | *P* | |
| Age |  |  | |  | | 11.6 | <0.001 |  |  | |  | -0.680 | | 0.497 | |
| Gender |  |  | |  | | 4.34 | 0.040 |  |  | |  | 0.420 | | 0.516 | |
|  | Male | 682 | | 102 | |  |  |  | 88 | | 94 |  | |  | |
|  | Female | 735 | | 79 | |  |  |  | 83 | | 77 |  | |  | |
| Residence | |  | |  | | 46.8 | <0.001 |  |  | |  | 0.110 | | 0.745 | |
|  | Urban | 1090 | | 96 | |  |  |  | 91 | | 94 |  | |  | |
|  | Rural | 326 | | 84 | |  |  |  | 80 | | 77 |  | |  | |
|  | Censor | 1 | | 1 | |  |  |  |  | |  |  | |  | |
| Family history | |  | |  | | 12.1 | <0.001 |  |  | |  | 1.260 | | 0.261 | |
|  | No | 1296 | | 151 | |  |  |  | 136 | | 144 |  | |  | |
|  | Yes | 121 | | 30 | |  |  |  | 35 | | 27 |  | |  | |
| BMI(kg/㎡) | |  | |  | | 49.3 | <0.001 |  |  | |  | 12.430 | | 0.002 | |
|  | <18.5 | 716 | | 118 | |  |  |  | 103 | | 109 |  | |  | |
|  | 18.5-23.9 | 75 | | 27 | |  |  |  | 10 | | 26 |  | |  | |
|  | ≥24 | 626 | | 37 | |  |  |  | 58 | | 36 |  | |  | |
| Alcohol drinking | |  | |  | | 25.5 | <0.001 |  |  | |  | 0.010 | | 0.908 | |
|  | No | 1163 | | 120 | |  |  |  | 121 | | 116 |  | |  | |
|  | Yes | 253 | | 61 | |  |  |  | 50 | | 55 |  | |  | |
| Tea drinking | |  | |  | | 40.3 | <0.001 |  |  | |  | 0.420 | | 0.516 | |
|  | No | 1055 | | 94 | |  |  |  | 85 | | 91 |  | |  | |
|  | Yes | 362 | | 87 | |  |  |  | 86 | | 80 |  | |  | |
| Tobacco smoking | |  | |  | | 17.7 | <0.001 |  |  | |  | 0.800 | | 0.371 | |
|  | No | 1048 | | 107 | |  |  |  | 111 | | 103 |  | |  | |
|  | Yes | 369 | | 74 | |  |  |  | 60 | | 68 |  | |  | |
| Total |  | 1417 | | 181 | |  |  |  | 171 | | 171 |  | |  | |
| The difference of age between case and control was evaluated by Chi-square test. Gender, age, residence, family history of cancers, BMI, tobacco smoking, tea, and alcohol drinking were tested by two-sample t-test. | | | | | | | | | | | | | | | |

| **Supplement Table 4** The cut-off values of rare earth elements | | |
| --- | --- | --- |
| Elements | Low level (μg/L) | High level(μg/L) |
| Cerium (Ce) | ≤1.071 | >1.071 |
| Praseodymium (Pr) | ≤0.022 | >0.022 |
| Neodymium (Nd) | ≤0.013 | >0.013 |
| Samarium (Sm) | ≤0.033 | >0.033 |
| Europium (Eu) | ≤0.005 | >0.005 |
| Dysprosium (Dy) | ≤0.019 | >0.019 |
| Scandium (Sc) | ≤6.715 | >6.715 |
| Lanthanum (La) | ≤0.246 | >0.246 |
| Yttrium (Y) | ≤0.258 | >0.258 |
| Thorium (Th) | ≤0.049 | >0.049 |
